# Functional gas exchange measures on ^129^Xe MRI and spectroscopy are associated with age, sex, and BMI in healthy subjects

**DOI:** 10.3389/fmed.2024.1342499

**Published:** 2024-04-08

**Authors:** David Mummy, Shuo Zhang, Aryil Bechtel, Junlan Lu, Joseph Mammarappallil, Suphachart Leewiwatwong, Anna Costelle, Aparna Swaminathan, Bastiaan Driehuys

**Affiliations:** ^1^Department of Radiology, Duke University, Durham, NC, United States; ^2^Medical Physics Graduate Program, Duke University, Durham, NC, United States; ^3^Department of Biomedical Engineering, Duke University, Durham, NC, United States; ^4^Department of Medicine, Duke University, Durham, NC, United States

**Keywords:** hyperpolarized ^129^Xe MRI, aging, pulmonary imaging, pulmonary spectroscopy, functional lung imaging

## Abstract

**Introduction:**

Hyperpolarized ^129^Xe MRI and spectroscopy is a rapidly growing technique for assessing lung function, with applications in a wide range of obstructive, restrictive, and pulmonary vascular disease. However, normal variations in ^129^Xe measures of gas exchange across healthy subjects are not well characterized, presenting an obstacle to differentiating disease processes from the consequences of expected physiological heterogeneity. Here, we use multivariate models to evaluate the role of age, sex, and BMI in a range of commonly used ^129^Xe measures of gas exchange.

**Materials and methods:**

Healthy subjects (*N* = 40, 16F, age 44.3 ± 17.8 yrs., min-max 22–87 years) with no history of cardiopulmonary disease underwent ^129^Xe gas exchange MRI and spectroscopy. We used multivariate linear models to assess the associations of age, sex, and body mass index (BMI) with the RBC:Membrane (RBC:M), membrane to gas (Mem:Gas), and red blood cell to gas (RBC:Gas) ratios, as well as measurements of RBC oscillation amplitude and RBC chemical shift.

**Results:**

Age, sex, and BMI were all significant covariates in the RBC:M model. Each additional 10 years of age was associated with a 0.05 decrease in RBC:M (*p* < 0.001), each additional 10 points of BMI was associated with a decrease of 0.07 (*p* = 0.02), and males were associated with a 0.17 higher RBC:M than females (*p* < 0.001). For Mem:Gas, male sex was associated with a decrease and BMI was associated with an increase. For RBC:Gas, age was associated with a decrease and male sex was associated with an increase. RBC oscillation amplitude increased with age and RBC chemical shift was not associated with any of the three covariates.

**Discussion:**

^129^Xe MRI and spectroscopy measurements in healthy subjects, particularly the widely used RBC:M measurement, exhibit heterogeneity associated in part with variations in subject age, sex, and BMI. Elucidating the contributions of these and other factors to ^129^Xe gas exchange measurements is a critical component for differentiating disease processes from expected variation in healthy subjects. Notably, the Mem:Gas and RBC chemical shift appear to be stable with aging, suggesting that unexplained deviations in these metrics may be signs of underlying abnormalities.

## 1 Introduction

^129^Xe gas exchange MRI and spectroscopy are emerging as techniques with clinical potential for evaluating disease status, progression, and therapy response in a range of cardiopulmonary diseases, including asthma (1–3), COPD (4, 5), COVID-19 (6, 7), interstitial lung diseases (8–11), and pulmonary hypertension (12–14). Inhaled ^129^Xe gas freely diffuses from the lung airspaces, through the alveolar-capillary interstitial membrane, and into the capillary bloodstream, where it transiently binds with red blood cells (RBCs). Three-dimensional images of ^129^Xe signal within each of these separate compartments (ventilation, interstitial membrane, and RBC) can then be individually reconstructed, enabling each of their contributions to overall gas exchange function to be quantified (15). The addition of whole-lung static and time-resolved magnetic resonance spectroscopy (MRS) provides insights into blood oxygenation and hemodynamics (13).

These imaging and spectroscopic techniques yield a range of measurements that reflect various aspects of cardiopulmonary function. On imaging, measurements of membrane and RBC signals are normalized to the spatially corresponding gas signal on a voxel-wise basis (termed the Mem:Gas and RBC:Gas ratios respectively). The resulting 3D gas, membrane, and RBC images are often quantified by the percentage of reduced or absent signal relative to a previously established reference population (16), and elevated membrane signal assessed using this method has been observed as a feature of interstitial lung diseases (9, 10). On spectroscopy, the widely used metric of the RBC to membrane signal ratio (RBC:M) reflects overall gas exchange efficiency (17). Additional emerging metrics of interest on spectroscopy are the RBC chemical shift, which may reflect the degree of delayed or impaired capillary blood oxygenation (18), and the amplitude of the dynamic RBC signal, which oscillates with the cardiac cycle (19) and is known to vary in a range of cardiopulmonary diseases (13).

The maturation of the field of ^129^Xe MRI/MRS and its ongoing transition to clinical use requires the development of suitable reference standards to (1) understand and interpret the findings for an individual patient, (2) understand the expected variation of these metrics in real-world populations, and thereby (3) differentiate potentially pathological findings from the expected heterogeneity across healthy subjects. Common ^129^Xe metrics like those cited above, which are used in research studies of patient populations, are often compared with samples of healthy subjects that are already on hand. However, those subjects may or may not be age-, sex-, or otherwise adequately matched with the population of interest. This raises the question of whether observed differences are due to disease or simply due to differences in basic subject characteristics. Specifically, variations in ^129^Xe gas exchange function in healthy subjects due to age, sex, and BMI are not well understood.

Recent work by Plummer et al. has sought to address this gap by modeling age-related changes in ^129^Xe image intensity histograms, with the goal of creating a tailored reference histogram for a given set of patient attributes (20). Here, we take an alternative approach (21) akin to that adopted by Collier et al. (22) and use multivariate linear models to evaluate age-related changes in ^129^Xe MRI and spectroscopy, including the RBC:M, RBC:Gas, Mem:Gas, RBC chemical shift, and RBC amplitude oscillations. Finally, we present a prototype of a “percent predicted” model for the RBC:M as an example approach for creating reference tables analogous to those used in conventional clinical pulmonary function tests (PFTs).

## 2 Materials and methods

### 2.1 Participants

Healthy subjects with no history of pulmonary disease, less than 5 pack-years smoking history, and no smoking history within the last 5 years were compiled from existing HIPAA-compliant ^129^Xe MRI studies at our institution (Pro00025110, Pro00059856, Pro00060259, Pro00106763, and Pro00107570). All participants underwent an informed consent process prior to recruitment and written consent at imaging. Hyperpolarized ^129^Xe was produced and administered under an investigational new drug approval (109,490). It was recently granted approval by the U.S. Food and Drug Administration for a ventilation indication (23).

### 2.2 ^129^Xe hyperpolarization and imaging acquisition

Hyperpolarized ^129^Xe was produced by continuous-flow spin-exchange optical pumping and cryogenic accumulation with commercially available hyperpolarizer systems (Models 9820 and 9810, Polarean) and dispensed into a Tedlar dose delivery bag (Jensen Inert Products). ^129^Xe MRI and spectroscopy were performed using a quadrature transmit-receive flexible vest coil (Clinical MR Solutions) on a 3 T scanner (Siemens, Trio/Prisma) during two separate breath holds. Participants were instructed to breathe out normally prior to inhaling the ^129^Xe dose, with the aim of beginning the inhale at approximately functional residual capacity (FRC). For calibration and spectroscopy, participants received a ^129^Xe dose equivalent (DE) of 81.9 ± 21.6 mL (mean ± sd), where the DE is the hypothetical volume of pure 100% hyperpolarized ^129^Xe that would produce the same net magnetization as the dose being sampled; this required an overall xenon volume of 420.6 ± 90.0 mL. For imaging, a larger DE of 177.6 ± 29.1 mL was used, corresponding to a xenon volume of 674.2 + 76.3 mL. For all doses, a nonpolarized ^129^Xe blend was added to increase the total delivery bag volume to approximately 20% of the subject’s forced vital capacity (FVC) per the Xenon Consortium protocol (24), or an untailored volume of 1 L (approximately one-third of the study population), with overall bag volumes of 943 ± 165 mL (min 500 mL, max 1,250 mL). Throughout imaging, heart rate and oxygen saturation were continuously monitored (Model 7500, Nonin).

Calibration and spectroscopy were performed during the first 10-s breath hold by acquiring ~500 ^129^Xe free-induction decays at 15-msec intervals (echo time, 0.45 msec; target flip angle, 20°; dwell time, 19 μsec; 512 points) (24). During the second 15-s breath hold, three-dimensional images were acquired using an interleaved radial acquisition of gas- and dissolved-phase (i.e., membrane uptake and RBC transfer) signals. Dissolved-phase images used a repetition time of 15 msec and a 20° flip angle [equivalent to an effective repetition time of 249 ms with a 90° flip angle (25)], matching the calibration scan, and an echo time of 0.45–0.50 ms chosen to target a 90° phase angle separation between the signals from the two compartments. The dissolved phase was excited using an RF pulse that was offset 218 ppm from the gas-phase peak.

### 2.3 Spectroscopy processing and analysis

Free-induction decays were acquired and fit to three peaks (gas, membrane uptake, and RBC transfer) in the time domain, with each peak described by amplitude, chemical shift, phase, and either one (gas and RBC) or two (membrane uptake) full widths at half maximum (19). The use of the two full widths for the membrane signal is due to its characterization as a Voigt line shape rather than the Lorentzian shape of the other two compartments. Static spectroscopy indices included the RBC:M and RBC chemical shift. In addition, the dynamic (time-resolved) spectroscopy measurement of amplitude of the RBC signal oscillations (“RBC amplitude oscillations”) was analyzed. These oscillations are driven by the cardiac cycle and were quantified by their peak-to-peak value relative to the mean (26).

### 2.4 Image reconstruction and analysis

The two dissolved-phase compartments were decomposed using the one-point Dixon method (15), using knowledge of the participant-specific RBC:M derived from spectroscopy. Three-dimensional images of the membrane and RBC compartments were individually reconstructed with a nominal isotropic resolution of 6.3 mm, and then divided by the gas-phase intensities from the same acquisition on a voxel-by-voxel basis to create maps relative to the gas signal. The resulting images were quantified using previously established methods (16). Only the ventilation image was corrected for coil transmit inhomogeneity using the N4ITK software tool (27). This correction was not needed for the Mem:Gas and RBC:Gas images since for such ratio maps, first-order coil inhomogeneity effects cancel out. The Mem:Gas and RBC:Gas images were characterized by calculating the mean of the individual voxel-wise ratios.

### 2.5 Quality control

We applied a qualitative rubric based on visual inspection of the images in conjunction with SNR, the presence of artifacts, adequate field of view, and so on. This quality control process was developed for a recent clinical trial (28) and a manuscript is in preparation describing our methods in detail.

### 2.6 Statistical analysis

Correlations between age and each individual metric were assessed using Spearman’s correlation. The RBC:M, Mem:Gas, and RBC:Gas ratios, as well as the RBC chemical shift and RBC amplitude oscillations, were each assessed individually using multivariate linear models, with age, sex, and BMI as covariates.

Finally, we created a prototype of a “percent predicted” model of RBC:Mem by using the parameter estimates from the corresponding multivariate linear model to predict the expected value for each individual patient based on their age, sex, and BMI and then dividing the observed value by this predicted value to yield the “RBC:M percent predicted” value (RBC:M%), analogous to the percent predicted values employed in PFTs. The resulting distribution of percent predicted values was assessed for normality with the Shapiro–Wilk test.

All statistical calculations were performed using R version 4.2.1.

## 3 Results

We recruited 40 total subjects (16F, age 44.3 ± 17.8 yrs., min-max 22–87 years) imaged between June 2018 and June 2022. Demographic, ^129^Xe gas exchange, and clinical data for this population are shown in Table 1. Subsets of these healthy subjects have also been described in previous studies (9, 29, 30).

**Table 1:** Demographic, pulmonary function test, and xenon MRI results for the study population, divided into three age groups.

	Age group		
	<30 (*N* = 13)	30–50 (*N* = 10)	>50 (*N* = 17)
Sex (F)	6 (46%)	6 (60%)	4 (24%)
Age	25.7 ± 2.6	37.8 ± 6.5	62.2 ± 9.6
BMI	25.4 ± 3.1	30.4 ± 5.9	25.0 ± 3.2
FVC%	102.6 ± 9.1	98.0 ± 20.3	109.4 ± 15.8
FEV1%	98.2 ± 12.8	99.4 ± 21.2	107.3 ± 16.2
FEV1/FVC%	98.0 ± 9.5	101.0 ± 8.9	98.7 ± 8.2
Mem:Gas × 100	0.71 [0.58, 0.83]	0.83 [0.75, 0.89]	0.66 [0.59, 0.68]
RBC:Gas × 100	0.45 [0.33, 0.46]	0.46 [0.33, 0.49]	0.32 [0.28, 0.38]
RBC:M	0.61 [0.51, 0.64]	0.49 [0.44, 0.55]	0.48 [0.44, 0.53]
RBC shift (ppm)	218.2 [218.1, 218.6]	218.2 [217.8, 218.3]	218.3 [218.2, 218.6]
RBC amplitude oscillations (%)	10.7 [9.8, 11.7]	10.4 [9.4, 11.7]	11.6 [9.9, 13.2]

Results are either mean ± standard deviation or median [first quartile, third quartile]. FEV1, forced expiratory volume in 1 s percent predicted; FVC%, forced vital capacity percent predicted; RBC:M, red blood cell to membrane ratio.

**Figure 1:**
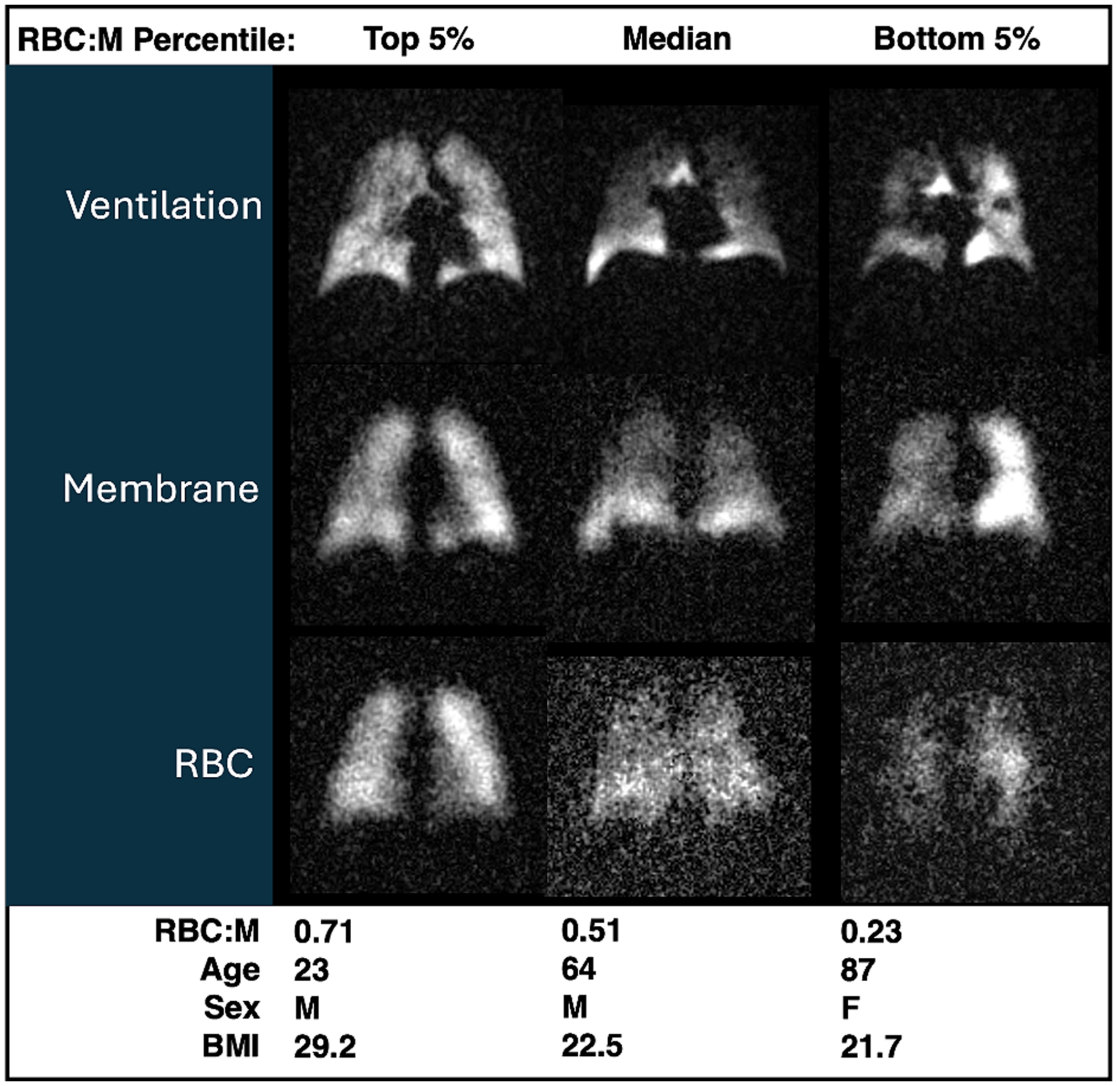
Grayscale images of ventilation, membrane, RBC from three participants with RBC:M values in the top fifth percentile (left), at the median (center), and in the bottom fifth percentile (right). Age, sex, and BMI are presented for each subject. Note that with increasing age, both RBC:M and the RBC signal decrease, while the membrane signal is relatively preserved. RBC, red blood cell; RBC:M, RBC to membrane ratio.

**Figure 2:**
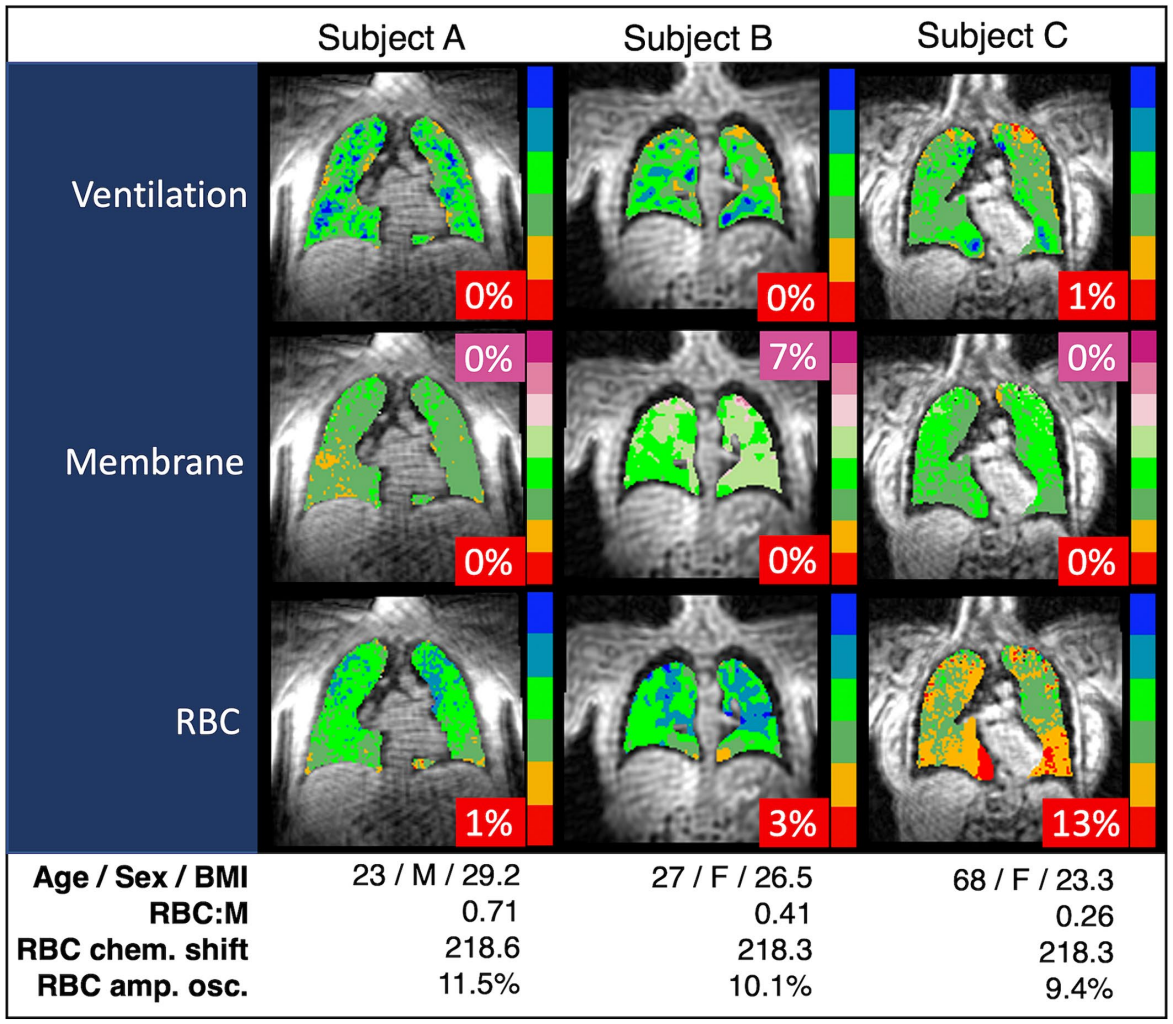
Spectroscopy measurements and color-binned ^129^Xe gas exchange images from three representative subjects. Subject A appears to have exemplary lung function with no abnormal values. Subject B has the increased membrane signal (purple, 7%) exhibited in some of the subjects with higher BMI, but otherwise exhibits normal lung function. Subject C has the RBC defects (red, 13%) and decreased RBC:M associated with age. In our binning method, “defects” are determined based on a healthy reference cohort used as a standard comparison for all subjects, and then interpreted on an individual basis using a similar approach to that presented in this work. Note that chemical shift is consistent across all three subjects. RBC:M, red blood cell to membrane ratio.

Representative grayscale images from subjects in the top fifth percentile of RBC:M, at the median value, and in the bottom fifth percentile are shown in Figure 1. Note that the age of the subjects increases with decreasing RBC:M, and the membrane signal is relatively preserved while the RBC signal shows a marked decrease with decreasing RBC:M. Representative samples of color-binned gas exchange imaging from three age groups are shown in Figure 2. One subject exhibits completely unremarkable findings; another subject exhibits modestly increased Mem:Gas, and another subject (age 68) shows marked RBC defects. On spectroscopy, while the RBC:M values exhibit a range of findings from normal to poor in these three subjects, the RBC amplitude oscillations are overall normal, and the RBC chemical shift shows minimal variation.

**Figure 3:**
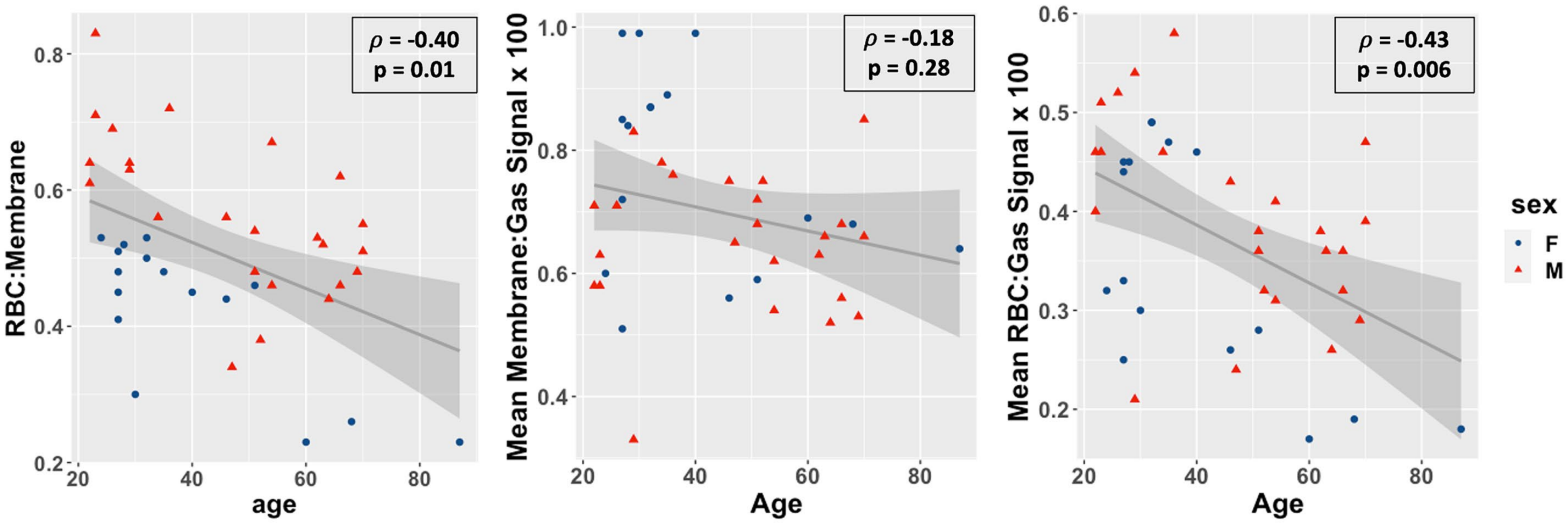
Scatterplots of the red blood cell to membrane ratio (RBC:M, left), mean membrane:gas signal (middle), and mean RBC:gas signal RBC amplitude oscillations (right) vs. age in our study population. Age was negatively correlated with RBC:M (r = −0.46, *p* = 0.003), not correlated with mean membrane signal (r = −0.19, *p* = 0.2), and negatively correlated with mean RBC signal (r = −0.43, *p* = 0.005). This result suggests that a decrease in RBC is the primary driver of the age-related decrease in RBC:M, rather than any meaningful changes in membrane signal. Note also the significant sex-related disparity in the RBC:M scatterplot, possibly driven in part by sex-related differences in hemoglobin.

**Figure 4:**
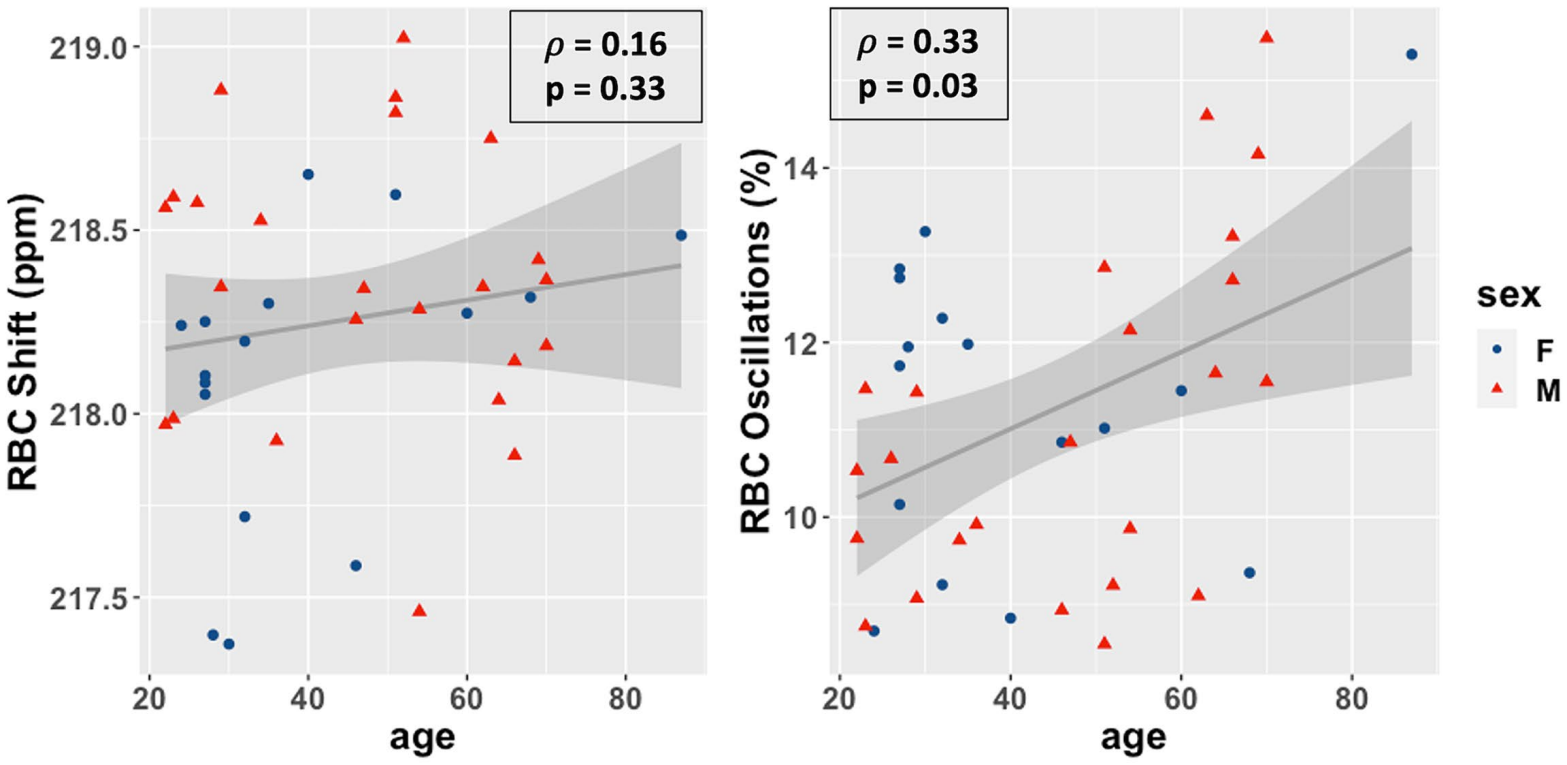
Scatterplots of the RBC chemical shift (left) and RBC amplitude oscillations (right) vs. age in our study population. Age was not correlated with RBC chemical shift (ρ = 0.15, *p* = 0.36) and positively correlated with RBC amplitude oscillations (r = 0.33, *p* = 0.03). RBC, red blood cell.

Overall, as shown in Figure 3, the RBC:M ratio was inversely correlated with age (Spearman’s ρ = −0.40, *p* = 0.01), as was the RBC:Gas (ρ = −0.43, *p* = 0.006), while the Mem:Gas was not correlated. As shown in Figure 4, the RBC chemical shift was not correlated with age, but RBC amplitude oscillations were correlated (ρ = 0.33, *p* = 0.03).

Results from all six of the multivariate models are shown in Table 2. In the model of RBC:M, all three parameters of age, sex, and BMI were significant. Specifically, each additional 10 years of age was associated with a 0.05 decrease in RBC:M (*p* < 0.001), male sex was associated with an increase of 0.17 (*p* < 0.001) vs. female, and each additional 10 points of BMI was associated with a decrease of 0.07 (*p* = 0.02). In the analogous model of Mem:Gas, males were associated with a reduction of 0.1 × 10^−2^ relative to females (*p* = 0.02), and every 10 points of BMI was associated with an increase of 0.1 × 10^−2^ (*p* = 0.03); Mem:Gas was not significantly affected by age. In the model of RBC:Gas, every 10 years of age was associated with a decrease of 0.03 × 10^−2^ (*p* < 0.001), and it was 0.07 × 10^−2^ higher for males than females (*p* = 0.02), while BMI was not a significant parameter. In the model of RBC amplitude oscillations, age was the only significant parameter (*p* < 0.01), with every 10 years of age associated with a 0.5% absolute increase in RBC amplitude oscillations. In the model of RBC chemical shift, none of the three parameters were significant.

**Table 2:** Multivariate model parameters in linear models of RBC:M, Mem:Gas, RBC:Gas, RBC oscillations, and the RBC chemical shift, with parameters with *p* < 0.05 shown in bold with an asterisk, with the exception of the intercept (italics), which was significant in all models.

Outcome	Covariate	Estimate	*p*-value
RBC:M	*Intercept*	*0.79*	*<0.001*
	Age	**−0.005**	**<0.001** ^*^
	Male sex	**0.17**	**<0.001** ^*^
	BMI	**−0.007**	**0.02** ^*^
Mean RBC:Gas ×100	*Intercept*	*0.5*	*<0.001*
	Age	**−0.003**	**<0.001** ^*^
	Male sex	**0.07**	**0.02** ^*^
	BMI	−0.0008	0.8
Mean Mem:Gas × 100	*Intercept*	*0.52*	*<0.001*
	Age	−0.0009	0.45
	Male sex	**−0.1**	**0.02** ^*^
	BMI	**0.01**	**0.03** ^*^
			
RBC amplitude	*Intercept*	*7.6*	*<0.001*
oscillations (%)	Age	**0.05**	**0.004** ^*^
	Male sex	−0.59	0.3
	BMI	0.07	0.3
RBC chemical shift	*Intercept*	*218.4*	*<0.001*
(ppm)	Age	0.002	0.7
	Male sex	0.24	0.07
	BMI	−0.014	0.3

Note the association of BMI with RBC:M and Mem:Gas, and the absence of any significant association between RBC chemical shift and age, sex, or BMI. RBC:M, red blood cell to membrane ratio.

**Figure 5:**
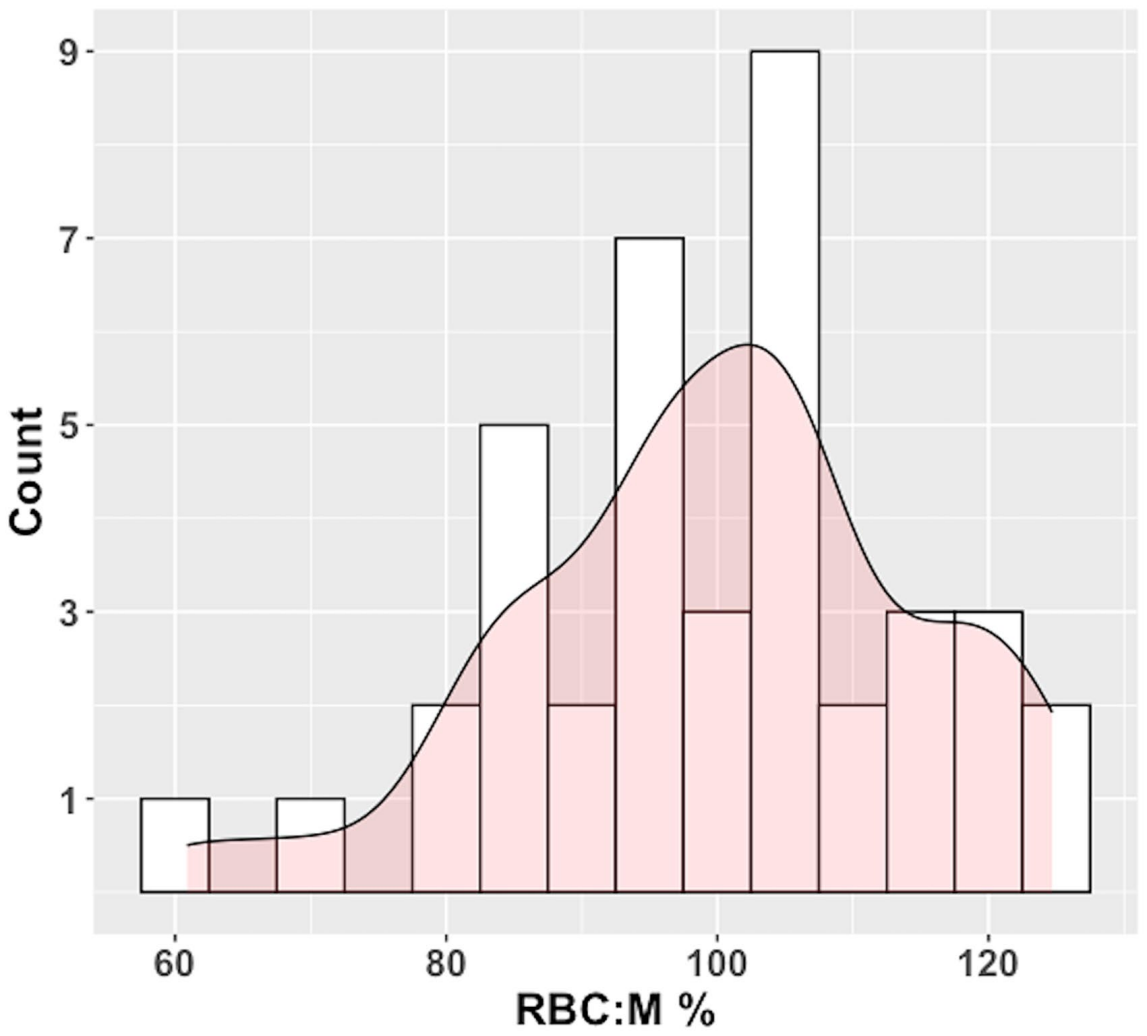
Histogram of RBC:M percent predicted (RBC:M%) values in our population with superposed curve fit. The distribution is not significantly different from normal (*p* = 0.45). Predicted values were determined by plugging age, sex, and BMI for each subject into the multivariate linear model in Table 2. Observed values for each subject were then divided by the predicted value to yield the %Pred value. The fifth percentile of the histogram is at an RBC:M% value of 78%, relatively close to the 80% percent predicted value generally used as a cutoff for “normal” in pulmonary function testing. RBC:M, red blood cell to membrane ratio.

The histogram of RBC:M percent predicted values based on the multivariate model of RBC:M as applied to our study population is shown in Figure 5, and the corresponding distribution of percent predicted values in this study population was not significantly different from normal (*p* = 0.45).

## 4 Discussion

This study suggests that age, sex, and BMI may all play roles of varying but critical significance in most measurements of ^129^Xe gas exchange. Thus, interpretations of findings in individual patients should be informed by knowledge of the specific effects of these characteristics. Notably, none of the attributes we studied appeared to significantly affect RBC chemical shift, suggesting that deviations in this metric may be a consistent marker of an underlying disease process.

We observed a clear age-related decrease in the RBC:M ratio, as also observed by Plummer et al. (20) and strikingly similar to the preliminary results reported by Collier et al. (22), who estimated an age coefficient of −0.004/yr. vs. our finding of −0.005/yr. As the name implies, the RBC:M depends on both the RBC and membrane signals, which obscures the contribution of these individual compartments; a decreased RBC:M could result from changes in the relative proportion of the two signals whether or not they individually increase or decrease. In this population, however, the stability of the membrane signal with age, coupled with the decrease in mean RBC signal, suggests that, rather than membrane diffusion limitation, vascular changes are the primary driver of age-related decreases in RBC:M. Both Plummer and Collier also observed an age-related decrease in Mem:Gas and RBC:Gas in adults, but differing methodologies across studies make these results more difficult to compare directly. Particularly, Plummer et al. included pediatric subjects in their study, and used a more complex curve-fitting technique to account for the non-linear behavior from child to adulthood.

The increased RBC amplitude oscillations with age may be caused in part by reduced pulmonary vascular volume, a known consequence of normal aging (31). This type of characterization may be useful to incorporate into models of pulmonary hypertension (19) or other studies that use xenon hemodynamics as an indicator of cardiovascular abnormalities. Ongoing work in our lab is advancing this work to understand the relationship between these measurements and cardiac output, and to derive fundamental cardiovascular parameters such as pulmonary vascular resistance from dynamic spectroscopy. It is also possible to derive 3D images of these temporal dynamics in the RBC signal using advanced reconstruction techniques, further refining the ability to characterize the spatial characteristics of dynamic cardiopulmonary function (12, 32).

The stability of the RBC chemical shift measurement with age, sex, and BMI is also noteworthy. This measurement is believed to reflect the efficiency with which capillary blood becomes fully oxygen-saturated as it passes through the gas exchanging region of the alveolus (9, 18, 19). This stability is somewhat non-intuitive, given that RBC defects increase with age, and prior work has shown in patients that such defects are associated with a reduced chemical shift (9, 19). This lower RBC shift has generally been attributed to reduced transit time through the gas exchange regions. Given that our work has shown RBC transfer defects to increase with aging, while RBC shift remains stable, this would suggest that cardiac output must decrease commensurately to the loss of capillary blood volume. While further work is needed to understand this behavior, the cardiac index is indeed known to decrease with age (33). Nonetheless, the fact that neither age, sex, nor BMI was significantly associated with RBC chemical shift changes would suggest that abnormal findings in this measurement may be an indicator of an underlying disease process independent of any these attributes.

The root cause of the association between BMI and membrane signal (both on its own in Mem:Gas and ostensibly in RBC:M, since we did not observe an association between BMI and the RBC signal) is not yet clear. However, a recent study in mice has shown that obesity is associated with a thickened interstitium and thus a thickened air-blood barrier (34). It is also possible that the increased membrane signal is due in part to a purely physical increased density of the lung due to the pressure of additional body weight, especially when the subject is in a supine position and tightly wrapped in a vest coil. Obesity has been associated with reduced FRC (35), and lung inflation volume alone is also known to affect ^129^Xe gas exchange measurements (36). Further work in this area is needed to isolate and test the effect of BMI on membrane signal separate from other potential factors such as lung inflation volume.

In our prototype of a “percent predicted” model of RBC:M, intended to be analogous to the familiar percent predicted tables used in pulmonary function testing, the percent predicted values were normally distributed, with a 5^th^ percentile value of 78%, very close to the 80% threshold generally used as a lower limit of normal for PFTs (although caution must be used when employing this type of fixed threshold to avoid over- or under-diagnosis) (37). This suggests the feasibility of this approach for creating age-normalized reference values to isolate true gas exchange abnormalities from the expected effects of aging both in individual subjects and across disease cohorts.

We acknowledge limitations to this work, particularly the need for a larger population evenly distributed across a wide range of age, sex, BMI, and other demographic characteristics to truly develop a reference cohort and expand our model to include additional variables and possible interaction terms. Future studies should also correct for hemoglobin, as this is known to have an effect on ^129^Xe gas exchange measurements and thus may be a confounder driving the apparent association with sex and RBC:M in our model due to the well-established sex-based differences in hemoglobin (29). However, hemoglobin values were only collected for a subset of our study population, thus limiting our ability to implement that correction in this work. In addition, our spectroscopic measurements may not be directly comparable with those of other groups, given that, rather than conventional phased Lorentzian fitting, we employ Voigt fitting of the membrane signal in the time domain to better represent the shape of the peak. We also note that despite upgrading our scanner during the study, we did not observe a discernable difference in SNR or signal quantification. We further note that different flip angles are applied to each compartment, and the effect of this relative flip angle difference in the context of constant replenishment of the dissolved-phase ^129^Xe signal is not well understood. However, in our experience, these differences are dwarfed by physiological and/or pathological variation and would require improved simulation, modeling, and experimentation to identify and tease out. Finally, we note that ^129^Xe MRI is currently a specialized and expensive technique, although with ongoing wider adoption, costs are expected to come down as economies of scale become possible. These limitations notwithstanding, this work represents a necessary first step toward establishing the feasibility of this approach to understanding age-related changes of widely-used xenon MRI gas exchange metrics and the development of generalized reference values.

In conclusion, hyperpolarized Xenon MRI reveals associations between age, sex, and BMI, particularly in the RBC:M ratio but also to varying degrees in the Mem:Gas, RBC:Gas, and RBC hemodynamic oscillations. These results suggest a clear need to incorporate the effects of these attributes when interpreting these metrics in individual patients or comparing across patients or populations. In contrast, none of these attributes appeared to affect the RBC chemical shift, and thus variations in this metric from the healthy reference may be a reliable marker of abnormal function. Further work in this area will determine the role of potential model covariates in a larger study population – particularly the potential role of BMI in the membrane signal – and refine our reference values and percent predicted models, with a goal of ultimately advancing clinical assessment and individual patient management using ^129^Xe MRI.

## Data availability statement

The raw data supporting the conclusions of this article will be made available by the authors, without undue reservation.

## Ethics statement

The studies involving humans were approved by Duke University Health System Institutional Review Board. The studies were conducted in accordance with the local legislation and institutional requirements. The participants provided their written informed consent to participate in this study.

## Author contributions

DM: Conceptualization, Data curation, Formal analysis, Investigation, Methodology, Writing – original draft, Writing – review & editing. SZ: Data curation, Formal analysis, Methodology, Software, Visualization, Writing – review & editing. AB: Data curation, Formal analysis, Software, Writing – review & editing. JL: Formal analysis, Software, Writing – review & editing. JM: Funding acquisition, Project administration, Supervision, Writing – review & editing. SL: Formal analysis, Software, Writing – review & editing. AC: Formal analysis, Methodology, Software, Writing – review & editing. AS: Conceptualization, Investigation, Methodology, Writing – review & editing. BD: Conceptualization, Data curation, Funding acquisition, Investigation, Methodology, Project administration, Supervision, Writing – original draft, Writing – review & editing.
